# Characterization of a candidate tetravalent vaccine based on 2'-O-methyltransferase mutants

**DOI:** 10.1371/journal.pone.0189262

**Published:** 2018-01-03

**Authors:** Roland Züst, Shi-Hua Li, Xuping Xie, Sumathy Velumani, Melissa Chng, Ying-Xiu Toh, Jing Zou, Hongping Dong, Chao Shan, Jassia Pang, Cheng-Feng Qin, Evan W. Newell, Pei-Yong Shi, Katja Fink

**Affiliations:** 1 Singapore Immunology Network, Agency for Science, Technology and Research, Singapore, Singapore; 2 Novartis Institute for Tropical Diseases, Chromos, Singapore, Singapore; 3 State Key Laboratory of Pathogen and Biosecurity, Beijing Institute of Microbiology and Epidemiology, Beijing, China; 4 Department of Biochemistry and Molecular Biology, University of Texas Medical Branch, Galveston, TX, United States of America; 5 Biological Resource Centre, Agency for Science, Technology and Research, Singapore, Singapore; 6 Department of Pharmacology and Toxicology, University of Texas Medical Branch, Galveston, TX, United States of America; 7 Sealy Center for Structural Biology and Molecular Biophysics, University of Texas Medical Branch, Galveston, TX, United States of America; Baylor College of Medicine, UNITED STATES

## Abstract

Dengue virus (DENV) is one of the most widespread arboviruses. The four DENV serotypes infect about 400 million people every year, causing 96 million clinical dengue cases, of which approximately 500’000 are severe and potentially life-threatening. The only licensed vaccine has a limited efficacy and is only recommended in regions with high endemicity. We previously reported that 2’-*O*-methyltransferase mutations in DENV-1 and DENV-2 block their capacity to inhibit type I IFNs and render the viruses attenuated *in vivo*, making them amenable as vaccine strains; here we apply this strategy to all four DENV serotypes to generate a tetravalent, non-chimeric live-attenuated dengue vaccine. 2’-*O*-methyltransferase mutants of all four serotypes are highly sensitive to type I IFN inhibition in human cells. The tetravalent formulation is attenuated and immunogenic in mice and cynomolgus macaques and elicits a response that protects from virus challenge. These results show the potential of 2’*-O*-methyltransferase mutant viruses as a safe, tetravalent, non-chimeric dengue vaccine.

## Introduction

Dengue virus (DENV) is a member of the *Flaviviridae* family transmitted by *Aedes* mosquitoes. Up to 80% of DENV infections are clinically inapparent [1]. However, at least every fifth infection causes dengue fever (DF) and the more severe forms of the disease, dengue hemorrhagic fever (DHF) and dengue shock syndrome (DSS). Four serotypes of DENV are known (DENV-1 to -4), each of which is capable of causing severe disease. The frequency, severity, and geographical spread of cases have increased over the past decades [2, 3]. In 2015, 3.2 million dengue cases were notified to the WHO by member states. An estimated 500’000 cases each year need hospitalization (http://www.who.int/mediacentre/factsheets/fs117/en/). Swift geographic expansion of *Aedes* mosquito habitats due to urbanization in Asia and South-Central America have accelerated the spread of dengue, resulting in a continuously increasing number of cases and putting half of the world's population at risk. Despite intensive global research efforts, only one licensed vaccine [4, 5] and no anti-viral treatment for dengue infection is available. Vaccine development has been hampered due to several reasons: (i) typically more than one serotype circulates in a region, therefore requiring that a vaccine protects against all four serotypes. (ii) A non-protective vaccination potentially increases the risk of vaccinees to develop the more severe forms of dengue during the next infection because of a known association of pre-existing immunity with severity [6–8]. (iii) Since most infections occur in developing countries, an ideal vaccine should be affordable and, due to logistic reasons, should be fully protective after one single administration [9–11]. Lastly, early vaccine research is complicated because (iv) no immune-competent animal model recapitulates human disease and (v) there are no defined correlates of protection.

The recently licensed tetravalent chimeric dengue-yellow fever vaccine (Dengvaxia ®) requires two booster injections and shows an overall efficacy of 65% against disease and 80% efficacy against hospitalization [12]. Several other vaccine candidates are being evaluated in clinical trials (reviewed in [13, 14]), among which two candidates (TV003 and TDV (formerly DENVax)) have begun efficacy trials [14].

Both the licensed vaccine Dengvaxia® and the candidates in advanced clinical testing (TV003 and TDV) are live-attenuated vaccines. Live attenuated vaccines are replication-competent viruses, which ideally induce immune responses and an immune memory comparable to those induced by wild-type viruses, without causing disease because of the low level of replication and hence low levels of inflammation. While Dengvaxia ® vaccine contains yellow fever virus (YFV) nonstructural proteins and does not code for any DENV non-structural (NS) proteins, TDV and TV003 contain DENV NS proteins for one serotype and three serotypes, respectively. NS proteins have been shown to be essential for the induction of an efficient CD8 T cell response, which seems important to mediate protection against DENV infection [15–18]. Live-attenuated vaccine approaches have been very successfully applied against two other members of the *Flaviviridae* family, YFV (YF-17D) and Japanese encephalitis virus (JEV SA14-14-2) [19].

Flaviviruses are positive-sense, single-stranded RNA viruses. The flavivirus genome encodes for three structural (C, prM, and E) and seven non-structural proteins (NS1, NS2A, NS2B, NS3, NS4A, NS4B, and NS5). NS5 is a multifunctional protein, consisting of the methyltransferase (MTase) activities responsible for 5’ RNA cap formation [20, 21] as well as internal RNA methylation [22] and the RNA-dependent RNA polymerase [23]. While N-7-methylation is essential for RNA translation and stability, 2’-*O*-MTase is not essential for viral replication in *vitro*, but protects the virus from the host’s innate immune system [24, 25]. Viruses that replicate in the cytoplasm, such as Flaviviruses, have evolved N7- and 2′-O-methyltransferases (MTase) to methylate their viral mRNA 5′ cap structures [26]. We previously have shown that DENV-1 and DENV-2 bearing mutations in the highly conserved MTase catalytic K-D-K-E tetrad are highly sensitive to IFN treatment and severely attenuated in mice and monkeys due to the inability of the virus to shield viral RNA from recognition by host innate immune factors [27].

Here we expand our research and additionally construct DENV-3 and DENV-4 MTase mutants and evaluate their properties *in vitro* and *in vivo*. We demonstrate that the mechanism of attenuation is the same as for DENV-1 and DENV-2 and we further show that a tetravalent formulation of DENV MTase mutants elicits a protective response in mice and monkeys. To our knowledge, this is the first tetravalent live-attenuated rational vaccine approach containing intact non-structural proteins sequences for each DENV serotype, therefore optimally activating the innate and adaptive immune response while being severely attenuated due to its susceptibility to the type I IFN response.

## Materials and methods

### Ethics statement

All experiments involving mice and monkeys were conducted according to the rules and guidelines of the Agri-Food and Veterinary Authority (AVA) and the National Advisory Committee for Laboratory Animal Research (NACLAR), Singapore. This study was approved by the Institutional Animal Care and Use Committee (IACUC) of BRC, IACUC Nos 090474 and 151018. Blood from anonymous healthy human donors was received from the blood bank at the National University Hospital of Singapore and blood donors gave written informed consent. In case the donors were minors, parents/guardians gave written informed consent on behalf of the child participant. Anonymous samples were used and the study was therefore exempted from full review by the Institutional Review Board of the National University of Singapore (NUS-IRB).

### Cells

BHK-21, C6/36, Vero and HEK-293 were purchased from the American type culture collection (http://www.atcc.org). HEK-293 and U937 cells expressing DC-SIGN were obtained by lentiviral transfection and subsequent cell sorting. All cells were maintained in minimal essential medium supplemented with fetal bovine serum (5%–10%). moDCs (verified by CD1a expression) were generated by isolating monocyte (CD14 positive selection kit from STEMCELL technologies) from PBMCs and culturing with IL-4 (200 IU/ml) and GM-CSF (granulocyte-macrophage colony- stimulating factor) (300IU/ml) for 5 days.

### Recombinant MTase preparation and methylation assays

The genes encoding the WT MTase domains of four serotypes (N-terminal 262, 292, 272 and 272 amino acids for DENV-1, DENV-2, DENV-3 and DENV-4) were amplified from corresponding virus strains and cloned into a pET-28a vector, respectively. Mutagenesis of MTase (K61A+E216A in DENV-1 strain West Pacific, and DENV-4 strain MY01-22713; K61A+E217A in DENV-2 strain TSV01 and DENV-3 strain VN32/96) was performed using QuikChange II XL site-directed mutagenesis kit (Stratagene). The complete sequence of each mutant MTase was verified by DNA sequencing. N7- and 2’-*O*-methylation assays were performed as described before [21]. The quantification of methyltransferase activity was performed using a phosphorimager (Typhoon FLA700, GE Healthcare Life Sciences).

### Preparation and characterization of recombinant DENV

Full-length infectious cDNA clones of DENV-1 (Western Pacific 74 strain), DENV-2 (TSV01 strain), DENV-3 (VN32/96), and DENV-4 (MY01-22713) were used to generate WT and mutant viruses. A standard mutagenesis protocol was used to engineer mutations into the MTase region as reported previously [21]. The protocols for *in vitro* transcription, RNA transfection, IFA, plaque assay, and growth kinetics were reported previously [28]. For challenge, strain 08K3126 (DENV-1, received from Environmental Health Institute EHI, Singapore [29]), strain D2Y98P (DENV-2, accession no. JF327392.1), strain VN32/96 (DENV-3, accession no. EU482459) and strain TVP360 (DENV-4, accession no. GU289913.1) were used.

### Mice

Female or male 6–8 week old IFN α/β/γ receptor deficient mice (AG129) were purchased from B&K Universal Limited with permission from Dr. M. Aguet (ISREC, School of Life Sciences Ecole Polytechnique Fédérale (EPFL)). All mice were bred and kept under specific pathogen-free conditions at the Biomedical Resource Centre, Singapore. All DENV used for immunization and challenge were produced in C6/36 cells. Animals were injected i.p. with a tetravalent MTase formulation (10'000 PFU of each serotype) or placebo (RPMI medium with FCS), respectively. At 30 days post infection, mice were challenged i.p. with DENV-1 (1x10^6^ PFU of strain 08K3126), DENV-2 (1x10^7^ PFU of strain D2Y98P), DENV-3 (1.5x10^6^ PFU of strain VN32/96) or DENV-4 (3x10^6^ PFU of strain TVP360). The amount of virus that generated a reproducible viremia in previous experiments in mice for each serotype was used.

### Cynomolgous monkey study

Six female cynomolgous macaques (CMs), weighing from 3.8 to 5.0 kg, were pre-screened for low IgG antibodies against DENV by ELISA. The animals tested negative for Herpes B virus (HBV), Simian Retrovirus (SRV), Simian T-Cell Leukaemia Virus (STLV) and Simian Immunodeficiency Virus (SIV) prior to entry into experiments. Animals were also negative for tuberculosis, MRSA (Methicillin-resistant Staphylococcus aureus), endoparasites and ectoparasites. CM were housed in cages that exceeded the space requirements recommended by the Guide for the Care and Use of Laboratory Animals (8^th^ edition) for Group 3 primates and rooms were on a 12:12 light:dark cycle, with temperatures maintained at 21–24°C and RH 30–70%. Animals were fed twice daily with Laboratory Fiber-Plus Monkey Diet 5049 (LabDiet, St Louis, MO) and filtered tap water was provided *ad libitum* by automatic watering devices. Enrichment in the form of food treats such as fresh fruit was provided daily and a variety of toys were also provided on a rotational basis. CM were group-housed for the entire duration of the study, except for one individual, ID-7056. ID-7056 displayed mild alopecia 15 days after the start of the study and was individually housed for veterinary observation. She was eventually re-housed with her original cage mates after one month and only separated for feeding thereafter.

Animals were fasted overnight and procedures conducted the following morning. Light sedation with ketamine (5–10 mg/kg) given intramuscularly (*i*.*m*., hindlimb or lumbar muscles) was performed for dosing, sample collection, and assessment of body temperature and weights. Animals were randomly divided into two groups and inoculated intradermally (i.d) into the left medial thigh with a tetravalent MTase formulation (10,000 PFU of each serotype) or placebo (RPMI medium with FCS), respectively.

Blood was collected from each CM on day 0, 1, 2, 3, and 6 post immunization to detect viremia. For neutralizing antibody tests, blood was taken 6 days before immunization (day -6) and on day 30 and 97 and post-immunization. On day 97 post-immunization, all animals were challenged by i.d. inoculation of 0.05 ml containing 10^5^ PFU of DENV-2 (D2Y98P) into the right medial thigh. Blood was collected on days 0, 1, 3, 4, and 7 for determination of viremia. U937-DC-SIGN cells were used to test the neutralizing capacity of plasma as described previously [30].

The animals were returned to the colony after the end of the experiments.

### Synthetic peptide library

Peptides were designed based on the sequence of DENV-1 strain TSV08-1 (accession nr. KR919821.1) and DENV-2 strain TSV01 and were purchased from Mimotopes (Australia). The peptide library consists of 396 DENV-1 and 392 DENV-2 15mer peptides overlapping by 10 amino acids and spanning the envelope, NS3, and NS5 sections. The purity of the peptides was above 80%, and their composition was confirmed by mass spectrometry analysis. Peptides were pooled according to their envelope, NS3, and NS5 categories. All peptides were dissolved in dimethyl sulfoxide (DMSO) at a concentration of 40 mg/ml, and intermediate working dilutions were performed in complete RPMI media (cRPMI: 10% FCS, 1×Penicillin, 1×Streptomycin, 1×L-glutamine, HEPES, and 1×β-mercaptoethanol).

### IFN-γ ELISPOT assay

Enzyme-linked immunosorbent spot (ELISPOT) assays for the detection of IFN-**γ**-producing cells were performed using the panel of DENV-1 and DENV-2 peptides. Assays were performed using thawed, *ex vivo*-isolated PBMCs. Briefly, 96-well plates (Multiscreen HTS; Millipore) were coated overnight at 4°C with 5 μg/ml of capture rat anti-human/monkey IFN-**γ** antibody (clone MT126L; Mabtech). Plates were washed with phosphate-buffered saline (PBS) and blocked with complete RPMI for 1 h at room temperature. The blocking solution was then removed. PBMCs were plated at 1×10^5^ and 5×10^4^ cells per well in the presence or absence of DENV envelop, NS3, and NS5 peptide pools at a concentration of 1 μg/ml in complete RPMI. For positive control wells, 5×10^4^ cells and 2.5×10^4^ cells were plated in the presence of 1 μg/ml anti-CD3 antibody (clone CD3-2; Mabtech). Cells were incubated for 16 h, after which plates were washed and 0.5 μg/ml of biotinylated anti-human IFN-**γ** (clone 7-B6-1; Mabtech) was added for 2 h at room temperature. After washing, 100 μl of streptavidin-alkaline phosphatase (Mabtech) diluted 1:2000 in PBS with 0.5% FCS was added and plates were incubated for 1 h at room temperature. Plates were washed and 50 μl of alkaline-phosphatase substrate BCIP-NBT plus (Mabtech) was added. After 10 to 15 min, the colorimetric reaction was stopped with running tap water. Spots were counted using an automated ELISPOT reader (Immunospot; Cellular Technology Limited). The number of IFN-**γ**-producing cells was expressed as spot-forming cells (SFC) relative to 1×10^5^ PBMCs. Values were calculated by subtracting the number of spots detected in the non-stimulated control wells. Values were considered positive if they were equal or greater than 5 spots.

### IFN pretreatment

Cells were seeded at 1 x 10^5^ per well in a 24-well plate and treated for 24 h prior to infection with medium or varying concentrations of human recombinant IFN-β (Immunotools). Cells were then infected at a multiplicity of infection (MOI) of 1 (HEK-DC) or 5 (moDCs) with WT or MTase mutant virus respectively, incubated for 48 h (moDCs) or 72 h (HEK-DC) and harvested and processed for flow cytometry as described below.

### Detection of infection by flow cytometry and flow cytometry-based neutralization assay

For determining the percentage of infected cells, cells were harvested, washed in PBS and fixed and permeabilized with Cytofix/Cytoperm (BD). Intracellular dengue E protein was stained with antibody 4G2 conjugated to Alexa 647 and fluorescent cells were measured by flow cytometry. Neutralization was measured as described previously [30].

### IgG ELISA

IgG ELISA was performed as described previously [30]. Briefly, 96-well polystyrene plates were coated with PEG-concentrated, UV-inactivated DENV or E protein that was produced in S2 cells as described previously [31]. Following washing, sera were diluted 1:50 in PBS-M, heat inactivated for 1 h at 55°C and threefold serial dilutions were added to the wells. Peroxidase-conjugated rabbit anti-mouse IgG followed and 3,3′,5,5′-Tetramethylbenzidine (TMB, from Sigma) as the enzyme substrate were used to detect dengue specific IgG. Endpoint titers were defined as the lowest dilution of plasma in which binding was twofold greater than the mean binding observed with the negative controls.

### Statistical analysis

Statistical tests were performed with GraphPad Prism software, using student's t test. The number of experiments, replicates (n) per group and variances (SD or SEM) are indicated in the figure legends.

## Results

### Methylation activities and replication competency of DENV1-4 MTase mutants

**Fig 1:**
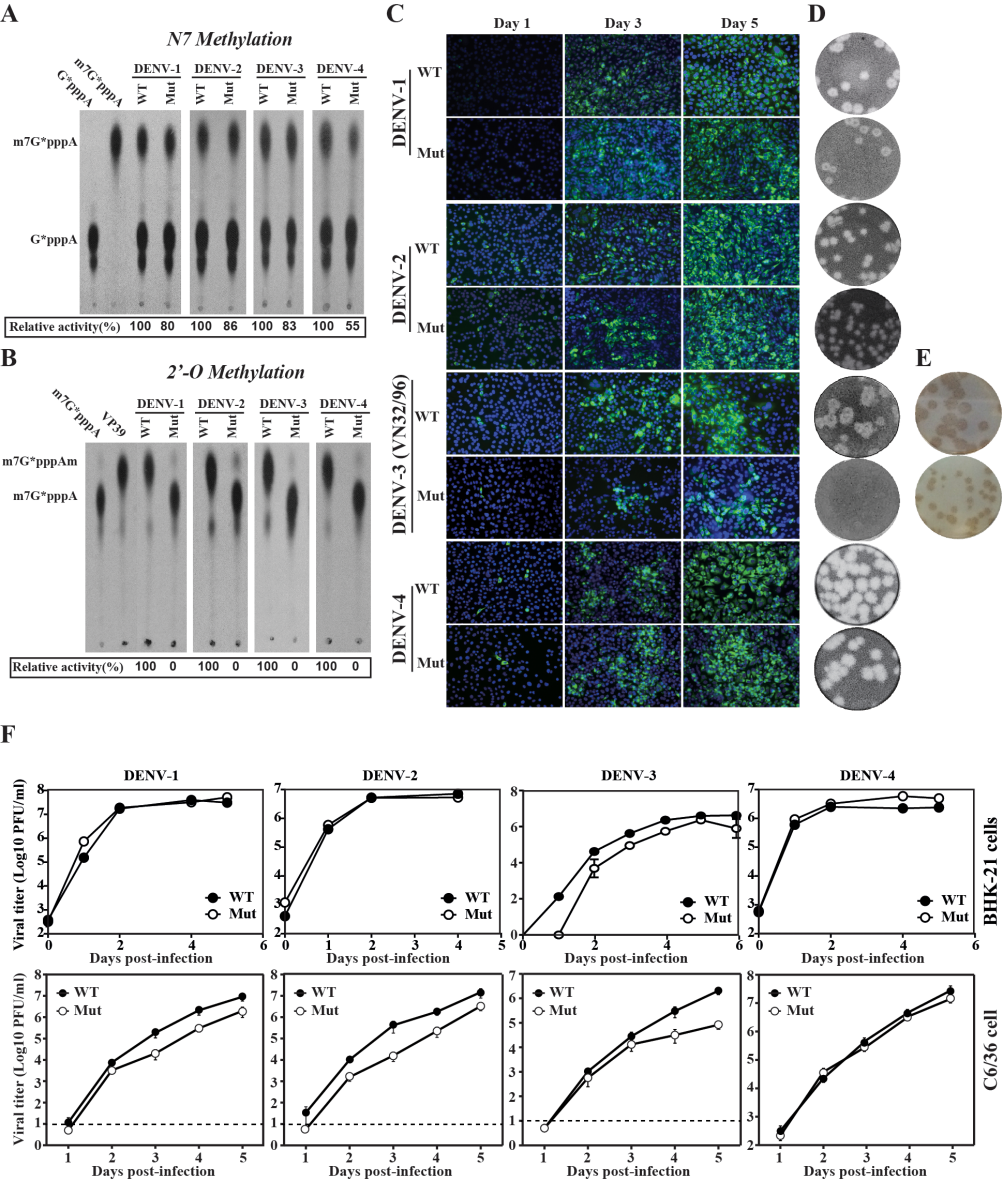
Characterization of NS5-Mtase mutants. A) Effects of MTase mutations on N7-MTase activities. B) Effects of MTase mutations on 2’-O-MTase activities. Recombinant wild-type (abbreviated as WT) and mutant (abbreviated as Mut). The mutant MTase of each DENV serotype contained double mutations within the K-D-K-E active site (with the underlined residues mutated to Alanine). MTases of all four DENV serotypes were assayed for GpppA-RNA→m7GpppA-RNA and m7GpppA-RNA→m7GpppAm-RNA conversions to indicate N7- and 2′-O-methylation activities, respectively. Relative methylation activities were indicated below each panel with WT activity set as 100%. C) Immunofluorescence analysis (IFA). BHK-21 cells were electroporated with equal amounts of in vitro transcribed WT and Mut genome-length RNAs of DENV-1 to -4. The mutant genome-length RNAs contained double mutations as described above. At indicated days post-transfection, intracellular E proteins were examined by IFA using mouse antibody 4G2 against DENV E protein and goat anti-mouse IgG conjugated with FITC as primary and secondary antibodies, respectively. D) Plaque morphology. WT and Mut viruses recovered from genome-length RNA-transfected cells were analyzed by standard CPE-based plaque assays using BHK cells. Plaques were developed on day 4 (DENV-2) or day 5 (DENV-1, -3 and -4) post-infection. E) Immunostaining. BHK-21 cells were infected with DENV-3 WT or Mut viruses harvested from genome-length RNA-transfected cells. On day 4 post-infection, cells were assayed by immunostaining using mouse antibody 4G2 against DENV E protein and goat anti-mouse IgG conjugated with horseradish peroxidase (HRP) as primary and secondary antibodies, respectively. F) Growth kinetics. BHK-21 and C3/36 cells were infected with WT and mutant viruses at an MOI of 0.01. Viral titers were measured at indicated time points using standard plaque assays (DENV-1, -2 and -4) or immunostaining (DENV-3). Average results and SD of three experiments are presented. Dash line indicates the limitation of detection (10 PFU/ml).

We have previously shown for DENV-1 and DENV-2 that mutation of the first Lys and the Asp of the tetrad K-D-K-E completely abolished 2’-*O*-MTase while maintaining substantial N7-methylation. To examine whether the same approach was feasible for all four serotypes, we cloned and expressed wild-type (WT) and K-D-K-E mutated (mut) recombinant MTase for DENV-1, DENV-2, DENV-3 and DENV-4, and examined the N7-methylation and 2’-*O*-methylation activities. While mutant enzymes retained 55–86% of the WT N7-methylation activity (Fig 1A), none of the mutants exhibited any 2’-*O*-methylation activity (Fig 1B). BHK-21 cells transfected with equal amounts of WT and mutant genome-length RNAs generated similar numbers of viral E protein-expressing cells, with the exception of mutant DENV-3, which appeared to infect less cells than DENV-3 WT (Fig 1C). To minimize reversion, each mutant genome-length RNA contained double amino acid mutations within the MTase K-D-K-E active site (with the underlined residues mutated to Alanine). WT and mutant RNAs from all four serotypes of DENV produced infectious viruses (passage 0), as quantified by plaque assays (Fig 1D) or immune-staining focus-forming assay (Fig 1E). The lack of plaque formation for DV3 mutant virus is likely due to the weak cytopathic effect on Vero cells upon infection (Fig 1D). However, immunostaining could reliably be used to measure the viral titer of this virus (Fig 1E). Sequencing of passages 0 and 5 from Vero cells showed that the engineered mutations were retained [32]. The replication of mutant viruses was not attenuated in hamster fibroblast cell line BHK-21 and was slightly attenuated in mosquito C6/36 cells for DENV-1 to -3, but not for DENV-4 (Fig 1F).

Collectively, the results demonstrated that the 2’-*O*-MTase mutants are suitable for amplification and consideration as DENV vaccine strains.

### DENV 2’-*O*-MTase mutants are highly sensitive to IFN-β pretreatment

**Fig 2:**
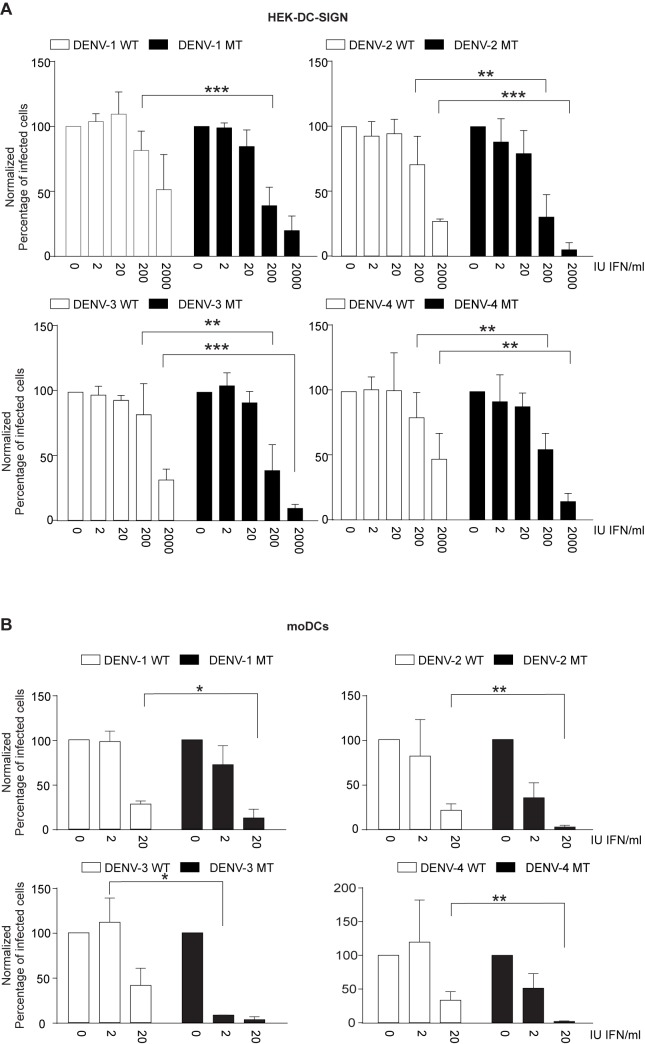
2’-*O*-MTase mutants are more sensitive to IFN. A) DENV WT or MTase mutant infection of HEK-DC-SIGN cells pretreated with increasing amount of IFN-β. HEK-DC-SIGN cells were seeded in a 24-well plate and after incubation overnight, pre-treated for 24 hours with the indicated amount of IFN- β. 24 hours after addition of IFN- β, cells were infected at an MOI of 1. B) DENV WT or MTase mutant infection of monocyte-derived dendritic cells (moDCs) pretreated with increasing amount of IFN-β. Means and SD are shown. Statistical analysis was performed using Student's t-test (****, p < 0.0001; ***, p < 0.001; **, p < 0.01; *, p < 0.05). Results shown are from three experiments with n = 4–11 measurements per condition (A) or from two experiments (from two different donors) with n = 4, except for DENV-3 where one experiment is shown from one donor with n = 2 (B).

We previously demonstrated that a DENV-2 MTase mutant has an increased sensitivity to IFN-β, which is partially mediated by IFIT1 [30]. To extend the results to all four DENV serotypes, we pretreated HEK cells expressing DC-SIGN, a receptor for dengue, with an increasing dose of IFN-β for 24 h and infected the cells with WT or mutant DENV. The MTase mutant viruses were significantly more sensitive to IFN-β pretreatment than the WT viruses (Fig 2A). Since antigen presenting cells such as dendritic cells are the host target cells of DENV at the physiological entry site of infection [33] we sought to test the IFN sensitivity of the mutants virus on monocyte-derived dendritic cells. As illustrated in Fig 2B, all four DENV serotype mutants can infect these primary human cells, but are sensitive to IFN.

### AG129 mice generate neutralizing antibodies against all four serotypes after tetravalent MTase mutant immunization and are protected against subsequent challenge

**Fig 3:**
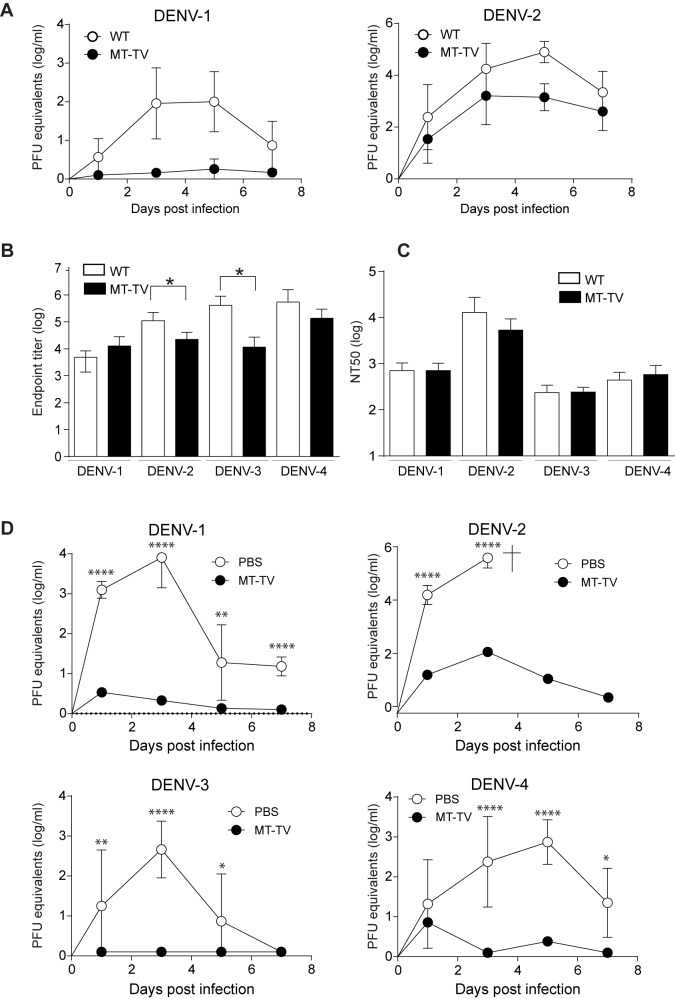
Dengue MTase mutants are attenuated and immunogenic in mice. A) Kinetics of MTase mutants and WT viremia *in vivo*. Mice were infected i.p. with 1×10^4^ PFU of WT virus or a mix of 1×10^4^ PFU of each MTase mutant (total of 4×10^4^ PFU). Viral titers in the plasma were measured at indicated time points by serotype-specific real-time RT-PCR. No virus was detected for WT and MTase mutant for DENV-3 and DENV-4, respectively. B) IgG titers of immunized mice. Blood was taken 30 days post immunization and total IgG antibody titers against DENV-1-4 were measured by UV-inactivated DENV particle ELISA. C) Neutralizing antibody (nAB) titers of imunized mice. Blood was taken 30 days post immunization and nAB against all four DENV serotypes were measured by flow cytometry-based neutralization assay. D) 30 days post immunization mice were challenged with DENV-1 (1x10^6^ PFU of strain 08K3126), DENV-2 (1x10^7^ PFU of strain D2Y98P), DENV-3 (1.5x10^6^ PFU of strain VN32/96) or DENV-4 (3x10^6^ PFU of strain TVP360). Viremia after challenge was measured by real-time PCR on days 1, 3, 5, and 7 post challenge. Naïve mice challenged with DENV-2 succumbed at day 4 post infection. Data are representative of three experiments with a total of 9–13 mice (A, B) or two experiments with a total of 6–9 mice per group (C, D). Shown are means with SD for all panels. Statistical analysis was performed using student's t-test (B, C, D), **** p<0.0001, *** p<0.001, ** p<0.01, * p<0.05.

To evaluate the MTase mutants as a tetravalent vaccine candidate, we immunized AG129 mice intraperitoneally (i.p.) with a mix of 10,000 PFU of each MTase mutant serotype or 10,000 PFU of WT virus individually from each serotype as control. Viremia was monitored over a period of seven days post infection using serotype-specific real-time PCR. DENV-1 and DENV-2 MTase mutants were attenuated in AG129 (Fig 3A). As expected, both WT and MTase mutant DENV-3 and DENV-4 were undetectable due to the low amount of input virus and due to the generally low virulence of these two serotypes in mice [34–36]. We next measured DENV-specific IgG titers and neutralizing antibody titers in plasma collected 30 days after immunization, using UV-inactivated DENV particle-coated ELISA plates and a U937-DC-SIGN based neutralization assay as readouts (Fig 3B and 3C). DENV-specific IgG antibody titers measured in ELISA were significantly higher for WT DENV-2 and DENV-3 compared to MTase mutant DENV-2 and DENV-3 but were similar for WT and mutant DENV-1 and DENV-4 (Fig 3B). No significant differences were observed between neutralizing antibodies induced by WT or TV MTase mutants (Fig 3C). Overall, the highest neutralizing antibody titers were generated against DENV-2, which correlated with higher viremia for DENV-2 compared to the other serotypes after immunization in mice. This trend was also observed when mice were immunized with a 10:1:10:10 formulation of DENV-1:DENV-2:DENV-3:DENV-4 (S1 Fig). To assess the protective capacity of the tetravalent formulation, mice were challenged 30 days post immunization with DENV-1 to -4 individually and viremia was used as readout to assess protection. Mice immunized with a tetravalent formulation of MTase mutants were protected against all four serotypes, whereas mice treated with PBS were infected consistently (Fig 3D). Notably, immunized mice were also protected against pathology and death caused by the lethal strain D2Y98P of DENV-2 used in this study.

### Macaques immunized with the tetravalent formulation of MTase mutants are protected after DENV-2 challenge

**Fig 4:**
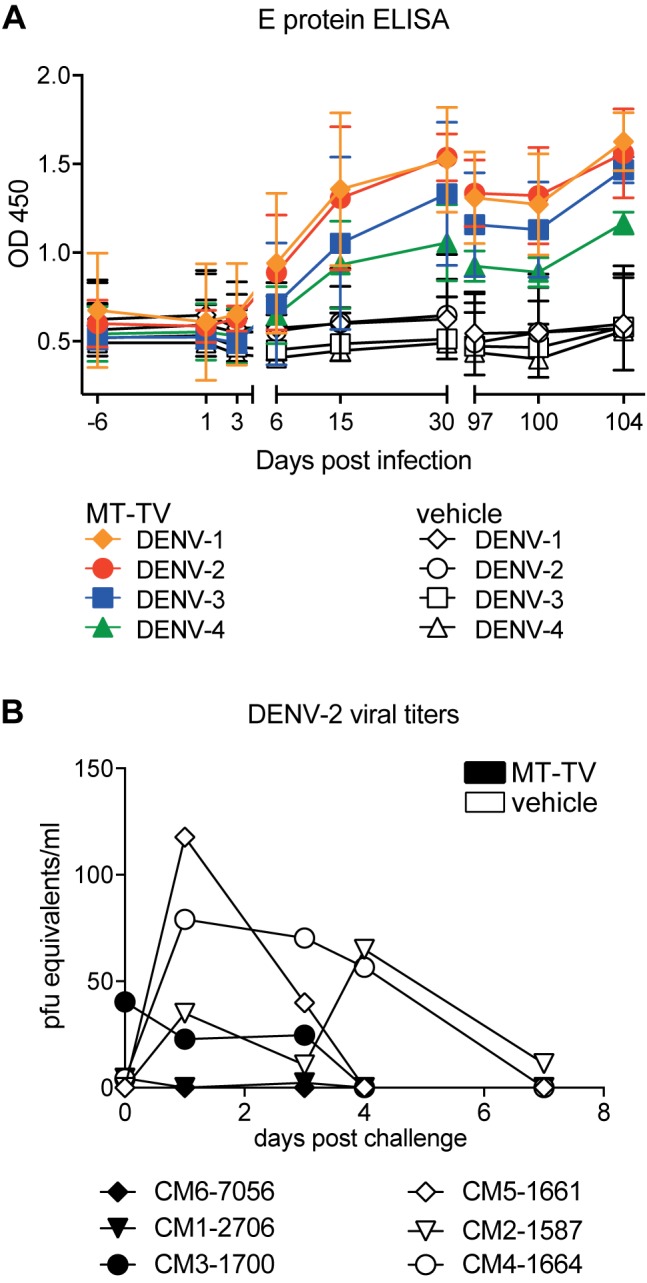
Dengue MTase mutants are immunogenic in monkeys and induce a protective immune response. A) Kinetics of DENV E protein-specific IgG titer of monkeys immunized with tetravalent MTase mutants formulation or RPMI placebo, respectively. Monkeys were immunized i.d. with a mix of 1×10^4^ PFU of each MTase mutant (total of 4×10^4^ PFU). B) Neutralizing antibody (nAB) titers of monkeys immunized as described above. nAB against all four DENV serotypes were measured by flow cytometry-based neutralization assay. C) 97 days post immunization monkeys were challenged with DENV-2 (1x10^5^ PFU of strain D2Y98P) i.d.. Viremia after challenge was measured by real-time PCR as indicated. Data are representative of one experiment with a total of 3 monkeys per group. Shown are means with SD for all panels. Statistical analysis was performed using Student's t-test (B). *, p < 0.05).

To assess the attenuation and efficacy of the tetravalent 2’-*O*-MTase mutant DENV vaccine approach in an immunologically competent host, two groups of three cynomolgus macaques (CM) each were immunized intra-dermally into the thigh with a tetravalent formulation of 10,000 PFU of each MTase mutant serotype (MT-TV) or medium, respectively. We chose the intradermal route because dermis and epidermis are rich in antigen-presenting cells, suggesting that delivery of vaccines to these layers should be more efficient [37], and physiologically relevant, given the normal mosquito-borne route of infection. Viremia was monitored on day 0, 1, 2, 3, and 6 after inoculation. No virus of any MTase mutant serotype could be detected by real-time PCR. All immunized monkeys developed antibodies to DENV-1 to DENV-4 from day 15 after immunization as analyzed by E protein ELISA (Fig 4A). Vaccinated monkeys showed neutralizing antibody titers against all DENV serotypes on day 30, with the strongest titers being induced to DENV-2 (Table 1). One monkey, CM6-7056, sero-converted partially to DENV-1 and DENV-2, whereas CM1-2706 converted partially to DENV-1, -2 and -4. CM1-2706 had a higher apparent titer for DENV-3 before immunization compared to after immunization. We would like to highlight that neutralizing titers in CM are relatively low in general compared to humans [27, 38]. Considering the error of neutralization assays and different baselines for different serotypes [39] CM1 probably did not respond to DENV-3. Neutralizing titers against all serotypes decreased until day 97 in all monkeys (Table 1). At day 98, vaccinated and placebo-treated monkeys were challenged with 100,000 PFU DENV-2, injected intra-dermally into the thigh contralateral to the immunization site. DENV-2 strain D2Y98P was chosen because it had been shown in a pilot experiment to consistently produce viremia in CM. Neutralizing antibodies increased after challenge with DENV-2, showing that the monkeys generated an anamnestic antibody response (Table 2). At day 3 post-challenge, vaccinated animals developed erythema that abated by the following day. No erythema was observed in placebo-treated animals. Two vaccinated animals, had visibly enlarged inguinal lymph nodes on the side of DENV injection (CM6-7056) and on both the sides, respectively (CM3-1700). The lymph node swelling disappeared within four days. Viremia was monitored by real-time PCR for 7 days post infection. Two vaccinated animals (CM6-7056 and 2706) were fully protected against DENV-2, whereas one-vaccinated individual (CM3-1700) showed barely detectable viral RNA (Fig 4B). In contrast, all non-vaccinated animals developed a viremia. Normal body temperature and no weight loss was observed for all animals throughout the study.

**Table 1:** Neutralizing antibody titers in CM before and after immunization with TV-MT.

	Day	Monkey ID			Mean NT_50_^
		CM1-2706	CM3-1700	CM6-7056	
DENV-1	-6	51.7	52.2	<20.0	**41.3**
	30	286.8	134.2	85.5	**168.8**
	97	29.5	31.0	<20.0	**26.8**
DENV-2	-6	28.7	23.8	28.1	**26.9**
	30	1006.0	1639.0	427.1	**1024.0**
	97	156.0	618.0	93.8	**289.3**
DENV-3	-6	130.7	60.9	*<20*.*0*	**105.1**
	30	120.1	119.5	30.6	**90.1**
	97	58.1	97.7	<20.0	**58.6**
DENV-4	-6	<20.0	<20.0	<20.0	**20.0**
	30	148.7	260.9	<20.0	**143.2**
	97	24.1	24.9	<20.0	**23.0**

^for mean calculation, values <20 were considered 20.

**Table 2:** Neutralizing antibody titers one day before challenge (day 97) and six days after challenge (day 104).

		TV-MT				Placebo			
NT50	Day	Monkey ID			Mean NT50	Monkey ID			Mean NT50
		CM1-2706	CM3-1700	CM6-7056		CM2-1587	CM4-1664	CM5-1661	
DENV-1	97	29.5	31.0	<20.0	**26.8**	<20	<20	<20	**20.0**
	104	267.8	184.8	176.5	**209.7**	28.6	53.66	140.5	**74.3**
DENV-2	97	156.0	618.0	93.8	**289.3**	74.0	20	135.7	**76.6**
	104	1519.0	4158.0	485.0	**2054.0**	305.1	381.6	188.4	**291.7**
DENV-3	97	58.1	97.7	20.0	**58.6**	<20	<20	<20	**20.0**
	104	629.0	262.3	78.4	**323.2**	127.8	125.3	139.1	**130.7**
DENV-4	97	24.1	24.9	<20.0	**23.0**	<20	<20	<20	**20.0**
	104	238.1	110.5	57.3	**135.3**	24.6	20	20	**21.5**

### T cell response in immunized monkeys

**Fig 5:**
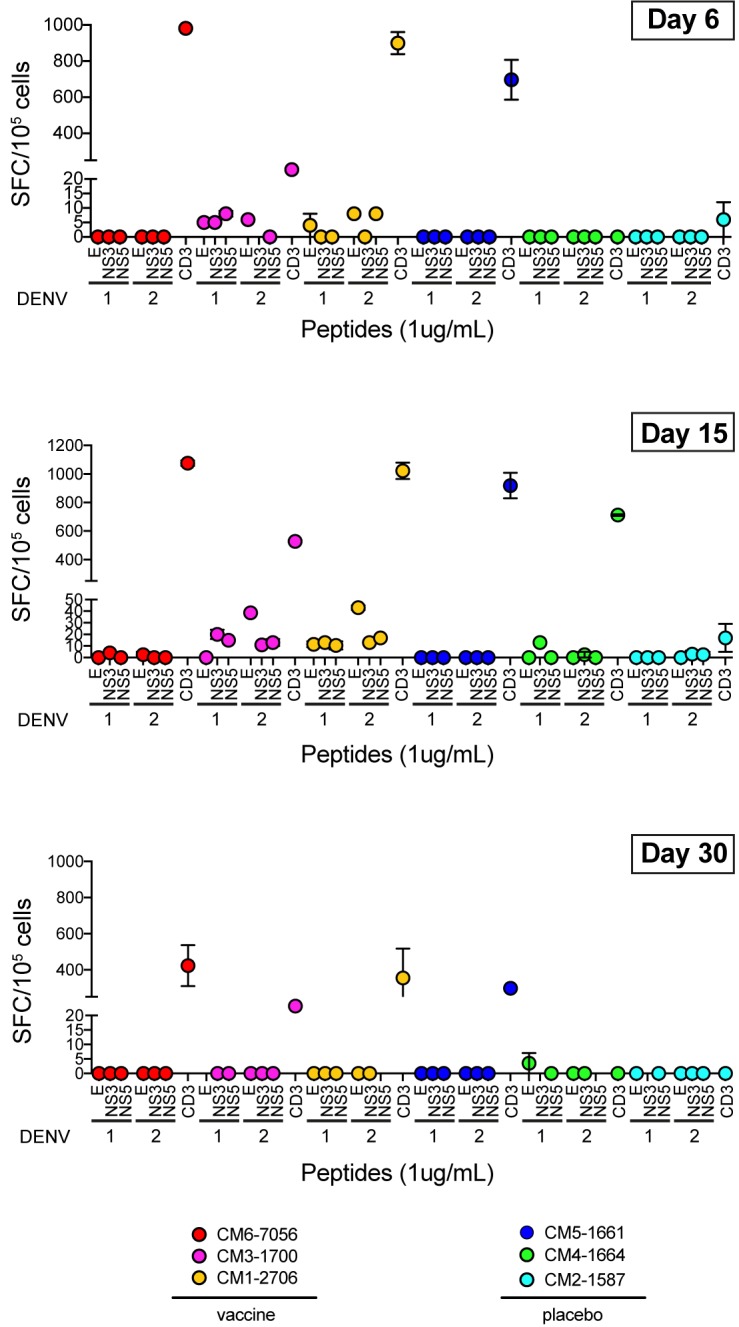
Dengue MTase mutants induce an E-protein targeted T cell response in CM. IFN-**γ** ELISPOT after overnight stimulation with peptide pools of E, NS3 and NS5 proteins of DENV-1 and DENV-2. PBMCs from three different time points after vaccination were tested. CD3 beads were used as positive control and values shown are after deduction of spots observed in the negative control. Mean and range of two wells tested per peptide pool is shown for each CM.

A potential advantage of non-chimeric DENV vaccine is the presence of DENV NS proteins from all four serotypes that can support the induction of a DENV-specific T cell response. To assess the magnitude and epitope preference of the T cell response in CM by IFN-**γ** ELISPOT, we re-stimulated PBMCs collected 6, 15, and 30 days after immunization with MT-TV or placebo with overlapping peptides of E, NS3, and NS5 proteins of DENV-1 and DENV-2. Two out of three vaccinated CM (CM3-1700 and CM1-2706) showed a specific response at day 15, with the highest number of T cells detected for E protein of DENV-2 and a lower response for NS3 and NS5 of both DENV-1 and DENV-2 (Fig 5). The T cell response was not detectable anymore by day 30, at least with the readout used in this study, suggesting that day 15 represented the peak of T cell expansion in vaccinated CM.

## Discussion

The better understanding of the mechanism of attenuation of 2’-*O*-MTase mutant flaviviruses has provided a novel approach for vaccine and antiviral development [24, 25, 40]. We showed previously in a proof-of-concept study that DENV-1 and DENV-2 MTase mutants are stable *in vitro*, and safe and immunogenic *in vivo* [27]. In this study, we extend our work to the tetravalent formulation of DENV MTase mutants, MT-TV.

Various dengue vaccine strategies are currently in development, including live attenuated virus, subunit vaccines, chimeric viruses, and DNA vaccines [13, 14, 41]. The YFV 17D-based chimeric dengue vaccine Dengvaxia® developed by Sanofi-Pasteur has been recently licensed in several countries [42]. However, the vaccine has an overall efficacy of about 60% and antibody titers appear to wane within a few years post immunization [12, 43, 44]. The reasons why Dengvaxia® did not generate protective immunity in particular in dengue-naïve individuals are not fully understood [45]. It can be speculated that the lack of DENV NS proteins in the chimeric construct might be one factor limiting the efficacy. CD4 epitopes are concentrated in the E protein, the capsid and the NS3 protein, whereas CD8 T cell epitopes are concentrated in the NS3 and NS5 proteins [16, 46, 47]. There is accumulating evidence that a functional T cell response is essential for protection against DENV infection [18, 47]. Besides Dengvaxia®, two other live attenuated vaccine candidates are currently being tested in efficacy trials, namely TV003 developed by the NIH and TDV developed by Takeda [14]. Both candidates are chimeric constructs, but an improved DENV-specific T cell response compared to Dengvaxia® is expected due to the expression of DENV NS proteins from at least one serotype. However, TV003 lacks NS proteins from DENV-2, while TDV lacks NS proteins from DENV-1, -3 and -4.

In the MT-TV vaccinated CM, we detected a higher T cell response to DENV E protein compared to NS3 and NS5, which might be suggestive of a dominant CD4^+^ T cell response. The ELISPOT readout used here does not distinguish between CD4^+^ and CD8^+^ T cells. The magnitude of the response with 10–50 spots/10^5^ PBMCs per DENV protein appeared relatively low. In comparison, the number of spots in human T cell ELISPOT for the same DENV proteins are in the range of <10 and 200 spots/10^5^ PBMCs during the post-febrile period, a time point comparable to day 15 assessed in CM in this study [46]. Further studies will be needed to investigate the T cell response to all four serotypes and to investigate whether CD4^+^ or CD8^+^ T cell responses are correlated with protection. It is interesting to note that CM6-7056, who had no detectable T cell response at day 15 (Fig 5), also showed the lowest NT50 titers in the vaccine group (Table 1).

An anamnestic antibody response was observed for all three vaccinated CM (Table 2). Interestingly, anamnestic responses were also reported in monkeys after previous infection with WT DENV [48]. Similarly, asymptomatic secondary or multiple infections in humans are common and normally detected by an increase of antibody titers indicative of anamnestic responses. This demonstrates that sterilizing immunity is not a prerequisite for protection from disease. While pre-existing neutralizing antibodies are essential for protection, it remains to be established which titer and which type of pre-existing antibodies are required for protection, and to which extent pre-existing antibodies can be complemented by a rapid anamnestic response to control the virus [49]. Interesting in this context, CM1700, who showed a detectable viremia after challenge, had the highest NT50 titers before challenge. This means that, similar to results from human challenge or prospective studies [50, 51], titers measured in the neutralization assay do not always correlate with protection. In both the mouse and CM model we observed an increased infectivity of DENV-2 compared to the other serotypes, for both WT and MT viruses. It is possible that this is due to a lower attenuation of DENV-2, or due to a generally higher susceptibility of mice and CM to DENV-2. It is unlikely that the lymph node swelling observed in two vaccinated CM after challenge is associated with the anamnestic antibody response since CM6-7056, who had the most obvious swelling, had the weakest initial response and the lowest fold increase in NT_50_ titer after challenge (tables 1 and 2). Part of the reaction could also be associated with the FCS present in the medium used for immunization and for challenge. Regardless, the LN response will have to be monitored carefully in future experiments.

Whether the balance between low virulence and high immunogenicity of 2’-*O*-MTase mutant viruses is achieved in humans remains to be elucidated. Given the fact that the MTase mutants retained the ability to infect antigen-presenting-cells (moDCs), we speculate that the candidate vaccine will induce an efficient immune response in humans. Our studies in human HEK293 and human moDCs show increased susceptibility of DENV MTase mutants to IFN-β *in vitro*, suggesting that the mutants will be attenuated in humans as well, and not only in mice and NHPs.

In this study we were limited to testing protection to DENV-2 in CM and protection to the other serotypes will have to be studied. It also remains to be seen whether and to which extent the strong immune response to DENV-2 in the mouse and CM experiments provided cross-protection to the other serotypes, instead of each serotype generating specific protective responses. While further work is needed to prove protection to all four DENV serotypes in non-mouse models, this is, to our knowledge, the first tetravalent live-attenuated rational vaccine approach containing DENV non-structural proteins from all serotypes and activating the innate and adaptive immune response while being severely attenuated due to its susceptibility to the IFN response.

## Supporting information

S1 Fig (PDF)

## Abbreviations

DENV: dengue virus
NHP: nonhuman primate
DHF: dengue hemorrhagic fever
DSS: dengue shock syndrome
TV: tetravalent
MTase: methyltransferase
