# Supplementary material for: Characterization of a candidate tetravalent vaccine based on 2'-O-methyltransferase mutants
Source: PLoS One. 2018 Jan 3;13(1):e0189262. doi: 10.1371/journal.pone.0189262 (PMC5751980; doi:10.1371/journal.pone.0189262)
Supplement: S1 Fig — (PDF) [file pone.0189262.s001.pdf]

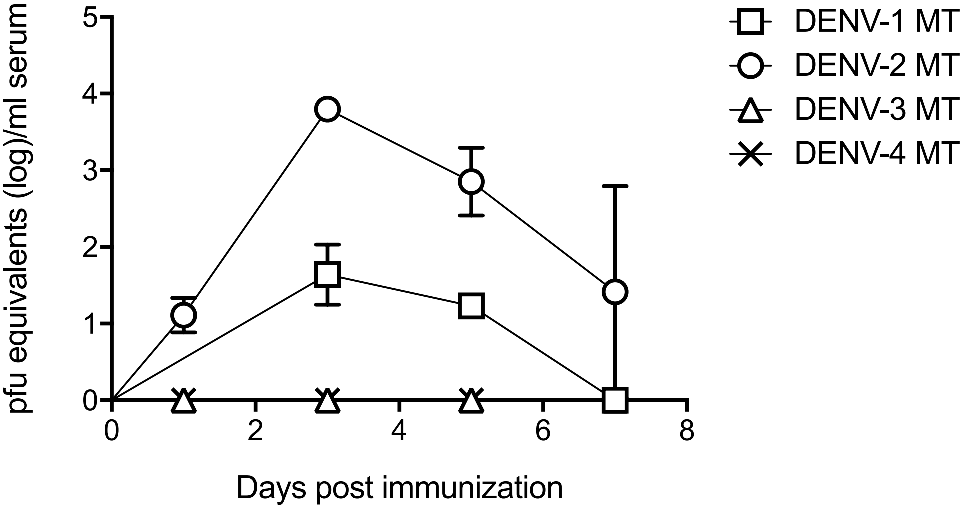

Supplementary Figure 1: Kinetcs of viremia after immunization with a DENV-1:DENV-2:DENV-3:DENV-4 ratio of 10:1:10:10. Mice (n=4 per group) were immunized with a mixture of 10^4 pfu of DENV-2 and 10^5 pfu of DENV-1, DENV-3 and DENV-4. Viremia was measured at the indicated time points after immunization by RT-PCR using serotype-specific primers. MT: 2'O-methyltransferase mutant virus. Means±SD are shown.
